# Use of antidepressant medication is associated with slower response to open-ended psychotherapy for depressed patients

**DOI:** 10.3389/fpsyt.2025.1503848

**Published:** 2025-09-01

**Authors:** Andreas Høstmælingen, Helene Amundsen Nissen-Lie, Jon Trygve Monsen, Erkki Heinonen, Bruce E. Wampold, Nikolai Czajkowski, Endre Visted, Bjørn Lau, Ole Andrè Solbakken

**Affiliations:** ^1^ Department of Psychology, University of Oslo, Oslo, Norway; ^2^ Department of Counseling Psychology, University of Wisconsin-Madison, Madison, WI, United States; ^3^ Department of Clinical Psychology, University of Bergen, Bergen, Norway

**Keywords:** depression, psychotherapy, antidepressant medication, interpersonal problems, open ended treatment

## Abstract

**Objective:**

Depressed patients often experience both symptomatic distress and interpersonal problems. Comorbid personality disorder (PD) has been shown to attenuate the benefits of psychotherapy. Also, antidepressant medication (ADM) may affect the response to psychotherapy. The objective of this study was to investigate changes in depressive symptoms and interpersonal problems for ADM users and non-medicated depressed patients during psychotherapy and follow-up, controlling for comorbid PD.

**Method:**

Depressive symptoms (SCL-90-R), and interpersonal problems (IIP-64) were assessed on 11 occasions for 166 depressed patients. ADM was used by 50.6% of the sample while 49.4% were unmedicated. Change during treatment and follow-up was assessed with multilevel modeling. We assessed whether ADM and PD predicted differences in symptom development.

**Results:**

Depressive symptoms significantly reduced at a rate of.05 per month in treatment (*p* <.001), corresponding to an effect size of 1.35. Interpersonal problems significantly reduced at a rate of.02 per month during treatment (*p* <.001), corresponding to an effect size of.47. There was no significant difference between ADM users and nonmedicated patients at baseline. ADM users had nearly twice as long treatment duration than nonmedicated patients, and ADM users had lower rate of symptom reduction than nonmedicated patients for depressive symptoms and interpersonal problems. There were no differences in rates of change between patients with and without comorbid PD.

**Conclusion:**

Medicated patients may experience less response to psychotherapy in terms of depressive symptoms and interpersonal problems compared to nonmedicated patients.

## 1 Introduction

Many patients are already being treated with antidepressant medication (ADM) when starting psychotherapy. It is however unclear how such ADM-use may affect psychotherapy outcomes. Research indicates combination treatment with psychotherapy and ADM has greater effects on symptom reduction relative to either treatment given separately (1–6). This could indicate that ADM adds to the effect of psychotherapy, thus providing a cumulative effect of two effective treatments. On the other hand, many patients experience negative side effects from ADM, such as sexual problems, weight gain, emotional numbness, and reduction in positive feelings (7–9). Accordingly, ADM may have paradoxical effects and contribute to worsening depressive symptoms (10, 11). Also, patients using ADM when starting psychotherapy have been found to experience less symptom improvement than nonmedicated patients during treatment (12) and during waitlist (13). Thus, ongoing treatment with ADM, particularly after long-term use, may cause a range of problems that may modify the clinical course and responsivity to subsequent psychotherapy (14), a phenomenon known as iatrogenic comorbidity (10). For instance, emotional blunting, experienced by 50% of ADM users (15), could negatively influence the ability to access emotions such as hurt and grief that need to be addressed in psychotherapy (16).

The presence of a personality disorder (PD) may also affect the response to psychotherapy for depression (17), which is relevant given the high prevalence of comorbid depression and PD (18). Depressed patients both with and without comorbid PD typically experience a range of interpersonal problems (18, 19), and research indicates depression and interpersonal problems mutually influence each other (20, 21). For instance, depressed patients may exhibit persistent reassurance seeking, resulting in significant others rejecting them, which in turn worsens depressive symptoms (21). High baseline levels of interpersonal problems have been found to negatively predict psychotherapy outcome for depression (22), and rate of improvement in interpersonal problems may play a crucial role in improving depressive symptoms (23). Thus, it is important to assess and monitor interpersonal problems for depressed patients seeking treatment. Additionally, as interpersonal problems are highly prevalent also for patients not having comorbid PD, it is important to control for potential confounding effects of comorbid personality disorder when assessing symptom change for both depressive symptoms and interpersonal problems during psychotherapy.

Previous research on psychotherapy outcomes has largely been conducted on patients with mild levels of psychopathology in university clinic samples (24). Thus, results from representative health care settings where patients often exhibit more severe psychopathology are needed. In this study we investigate symptom change for ADM users and non-medicated depressed patients during treatment and follow-up of open-ended psychotherapy for depressive symptoms and interpersonal problems. The study was conducted using a sample from a naturalistic study of open-ended psychotherapy under clinically representative conditions. Specifically, we investigated two research questions: 1) Do patients experience an overall decrease during open-ended psychotherapy and follow-up for depressive symptoms and interpersonal problems? 2) Are there differences in symptom change between non-medicated patients and ADM users during treatment and follow-up, controlling for comorbid PD?

## 2 Method

### 2.1 Study overview

This study is a quasi-experimental study based on data from the Norwegian Multi-Site Study of Process and Outcome in Psychotherapy (NMSPOP; 25). In-depth descriptions of the study are provided elsewhere (24, 26). NMSPOP is naturalistic, practice-oriented, outpatient psychotherapy study following 370 patients treated at eight different sites within the public health care system in Norway. Data were collected from 1995 to 2008. Trained coordinators at each site recruited participants to the project. They were instructed to select patients randomly from the caseload, but no formal randomization procedure was applied. After a thorough assessment, trained psychotherapists who had agreed to participate in the study, offered open-ended individual psychotherapy to the patients recruited, while one site gave time-limited (40 session) therapy. The treatments offered were non-manualized and representative of psychotherapy as conducted within a public outpatient setting with either clinical psychologists, psychiatrists, psychiatrists in training, psychiatric nurses, or clinical social workers, each with specific psychotherapy training. Initial diagnostic assessment was conducted by clinical psychologists or psychiatrists using SCID 1 & 2 (27, 28) based on the diagnostical and statistical manual IV (29). Patients who had a primary diagnosis of ongoing psychosis, drug/alcohol abuse, or mental retardation (IQ < 70) were excluded. Exclusion criteria also included ages under 18 years, and need for emergency hospitalization.

### 2.2 Participants

For the present study we studied a subsample (n = 184) of the NMSPOP sample diagnosed with a depressive disorder. As we wanted to compare patients using ADM to non-medicated patients, patients who were not using ADM but used other psychotropic medication (i.e., antipsychotics, anxiolytics, antiepileptics, hypnotics) during treatment were excluded from analysis. The 18 patients using medication not classified as ADM were excluded, leaving a final sample of N = 166. Comorbid axis 1 disorders were anxiety disorder (66.3%, n = 110), somatoform disorder (22.9%, n = 38), eating disorder (10.2%, n = 17), and substance abuse disorder (2.4%, n = 4). Approximately half (54.8%, n = 91) of the sample met criteria for at least one personality disorder (52.7% cluster C, 35.2% cluster A, 8.8% cluster B). Approximately half of the patients (55.4%, n = 92) had previously sought help within the last two years prior to inclusion and reported having had a mean (M) duration of problems for more than 15 years (*M* = 15.55 years, *SD* = 19.6*)* before seeking help. The sample also had a long history of previous treatment attempts having on average sought help six times (*M* = 6.4, *SD* = 8.7*)* from general practitioners. The mean age was 34.8 years (*SD* = 9.2), 72.9% (n = 121) were female, 50.6% (n = 84) were married or co-habiting, 66.9% (n = 111) had children, and 33.1% (n = 55) had higher education (i.e., bachelor or above). At onset, 63.3% (n = 105) had regular employment more than 15 hours per week, and 40.9% (n = 68) were full time employed or studying. 75.3% (n = 125) stated they usually had enough money to support themselves. Thus, the patient sample was heterogenous with many patients displaying moderate to severe pathology, with high degree of comorbidity, a long history of distress and several previous treatment attempts.

### 2.3 Treatment and therapists

**Figure 1:**
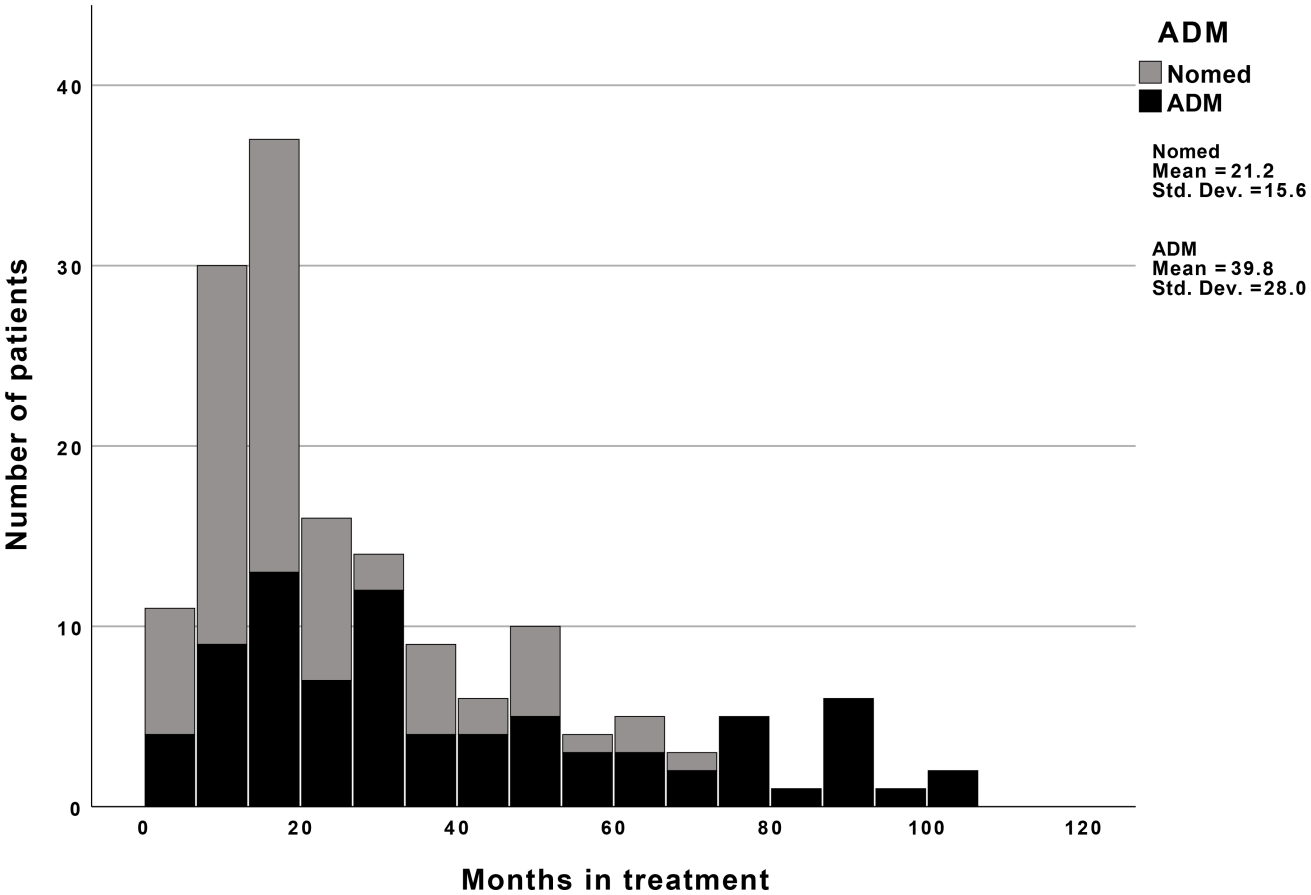
Months in treatment for nonmedicated and medicated patients. Nomed, Nonmedicated patients; ADM, Patients using antidepressant medication.

The treatments were open-ended and non-manualized. Each patient/therapist pair was instructed to reach an agreement as to when treatment should end based on their joint appraisal of the progress made in therapy. The therapists (N = 62) participating in the project were ordinary staff members at the public outpatient clinics, as well as some graduate psychology students under supervision at a university student clinic. The therapist sample consisted of clinical psychologists (58.1%), graduate psychology students (9.7%), psychiatrists or psychiatry trainees (16.1%), social workers (6.5%) and others (9.6%). Of the therapists, 82% reported to have a salient psychoanalytic/psychodynamic orientation, but salient orientations for humanistic (29%) and cognitive (27%) were also reported. Their mean level of experience practicing psychotherapy was 9 years (*SD* = 7.05). For the entire sample, the mean treatment duration was 30.6 months (*SD* = 24.5), ranging from 3 to 103.7 months for the entire sample. The mean number of sessions was 64.4 (*SD* = 74.1), ranging from 2 to 364 sessions. Non-medicated patients had a mean treatment duration of 21.2 months (*SD* = 15.6), with a range from 3 to 69.5 months, and a mean number of 44.3 (*SD* = 53.6) sessions ranging from 2 to 315 sessions. ADM users had a mean treatment duration of 39.8 months (SD = 28.0), with a range from 4 to 103.7 months, and a mean number of 84.1 (*SD* = 85.5) sessions ranging from 2 to 364 sessions. Of the patients using ADM, the mean treatment duration for patients who did not use ADM at start of treatment but began during treatment was 44.6 (*SD* = 27.9) months. For patients starting psychotherapy with simultaneous ADM use and quit ADM during, the mean treatment duration was 38.8 (*SD* = 27.0) months, while treatment duration for patients using ADM at both start and end was 37.7 (*SD* = 29.8) months. See **Figure 1** for a histogram of the frequency distribution of months in treatment.

### 2.4 Medication

Medication was classified according to the WHO Anatomical Therapeutic Chemical (ATC) Classification (30). Patients using ADM (ATC-code N06A) at any point during treatment were classified as ADM-users (coded 1) and compared to patients who did not use any medication during treatment (coded 0). Of the total sample of 166 patients, 82 patients (49.4%) were unmedicated, while 84 (50.6%) were using at least one type of ADM during treatment. Of the 82 patients who were unmedicated during treatment, 23 (28%) had received at least one course of ADM during the two years prior to treatment. Of the 84 patients who used ADM during treatment, 54 (64.3%) had received at least one course of ADM during the two years prior to treatment. Of the 84 patients using ADM 29 (34.5%) started and ended treatment using ADM, 35 (41.7%) started treatment using ADM but not at the end, and 20 (23.8%) did not use ADM at start but did at the end of treatment.

### 2.5 Procedures

Trained coordinators, either a clinical psychologist or psychiatrist at each treatment clinic, were responsible for recruitment of participants to the project. Coordinators were instructed to select patients from their caseload randomly, but no formal randomization procedure was applied. Patients were assigned to a therapist based on the therapists’ availability. Diagnostic evaluations were done according to the Diagnostic and Statistical Manual of Mental Disorders, fourth edition (29) and use of the Semi-structured Clinical Interviews for DSM-IV for Axis I and II (SCID I & II; 27, 28) and conducted by clinical experts (clinical psychologists and/or psychiatrists) at each site. Diagnostic evaluations were conducted at start and termination of therapy.

### 2.6 Outcomes and measures

The outcomes were the depression scale on Symptom Check List-90-Revised (SCL-90-R; 31), and the Inventory of Interpersonal Problems-64 (IIP-64; 32). Patients completed these questionnaires pre-treatment (T1), at sessions 3, 12, 20, 40, 60, and 80, after treatment termination (T2), at six-month follow-up (T3), one-year follow-up (T4), and 2.5-year follow-up (T5).

The SCL-90-R is a broad measure of symptom distress consisting of 90 items with each item scored on a Likert scale from 0-4. It produces nine symptom specific subscales, and three global indexes of symptom severity (31). The Norwegian version of SCL-90-R has demonstrated good psychometric properties (33). The depression scale of SCL-90-R (Dep-SCL-90-R) consists of 13 items scored on a Likert scale from 0–4 and is calculated by dividing total sum score (range 0-52) by number of answered items, resulting in a score between 0 and 4 (31). The Dep-SCL-90-R covers depression symptoms such as low mood, loss of interest, low energy, feelings of self-blame and worthlessness, worrying and hopelessness, and thoughts of self-harm and suicide. The Norwegian version of Dep-SCL-90-R has demonstrated high internal consistency with Cronbach’s alphas of.91 (34) and.89 (35) in non-clinical samples, and.88 in clinical samples (36). Dep-SCL-90-R has also demonstrated high concurrent validity (.72) with other measures of depression (Hospital Anxiety Depression Scale - Depression; 37). In the present study, Cronbach’s alpha of the SCL-90-R depression scale was.83.

IIP-64 is a broad measure assessing a variety of interpersonal problems, consisting of 64 items scored on a Likert scale from 0-4. The IIP-64 yields eight octant sum scores, indicating specific domains of interpersonal functioning and one global score (32). In the current study, we used the global score which is calculated by dividing the total sum score (range 0-256) by the number of items, resulting in a score between 0 and 4. This global score of the IIP-64 has been consistently linked to symptom severity (38), and IIP-64 has demonstrated good convergent validity, test-retest reliability and internal consistency (32, 39). In the present study, Cronbach’s alpha of the IIP-64 global index was.92.

### 2.7 Statistical procedures

#### 2.7.1 Data structure

As repeated measurements were nested within patients, we assessed symptom development using multilevel modeling (MLM; 40). Each patient was assessed at 11 occasions (i.e., start of treatment (T1), 3^rd^, 12^th^, 20^th^, 40^th^, 60^th^ and 80^th^ sessions, as well as end of treatment (T2), six month follow up (T3), one-year follow-up (T4) and two-and-a-half-year follow-up (T5)). For multilevel analyses of repeated measures it is important to first model time (41) before entering predictors. We treated time as a piecewise linear variable in the multilevel models, defining separate slopes for the treatment period (i.e., months T1 to T2) and for the follow-up period (i.e., months T3 to T5), using the analytic approach described by Bauer and Curran (42). The first slope (“treatment”) was equal to the months when the patient was in treatment but stopped incrementing forward when treatment was terminated (i.e., after T2). The second (“follow-up”) was set to be zero when the patient was in treatment and incremented forward after treatment termination. Thus, “treatment” and “follow-up” were used as variables which could display different symptom slopes in the subsequent multi-level model analyses. As some patients could have a short duration of treatment (e.g., treatment concluded after 3 or 12 sessions), some occasions were estimated (see supplemental materials and **Supplementary Table S1** for an illustration of the data structure).

#### 2.7.2 Multi level model

For all models, the intercept was centered at T1. Time was coded as months. Model fit was compared using –2 log likelihood (-2ll), and likelihood ratio tests (LRT) were conducted to assess improvement in model fit for each step for nested models. The models were tested using a variance components (VC) covariance structure for models with only fixed effects, and unstructured (UN) covariance structure for models with random effects and interactions. For estimation of regression coefficients and variance components, full maximum likelihood (FML) was used (41). For each of the two outcomes (i.e., Dep-SCL-90-R, IIP-64) a multilevel model was fitted for the two-phase piecewise model (treatment, follow-up). **Tables 1** and **2** present each step for the model building of the MLMs. Each model was built in six steps following Hox et al. (41). Since fixed parameters are typically estimated with more precision than random parameters (41), the model building steps were conducted such that fixed parameters (i.e., means) were estimated first. Then random parameters (allowing slopes to vary between individuals) were added before interaction effects were added last.

**Table 1:** Results from multilevel analysis of development of depressive symptoms (depression scale of SCL-90-R) during treatment and follow-up.

Model	Model 0: Intercept only	Model 1: + time	Model 2: + ADM, PD	Model 3†: + time random	Model 4†: + interaction PD	Model 5†: + interaction ADM
Fixed part	Est. (s.e.)	Est. (s.e.)	Est. (s.e.)	Est. (s.e.)	Est. (s.e.)	Est. (s.e.)
Int.	1.68 (.053)***	1.89 (.055)***	1.56 (.086)***	1.75 (.085)***	1.78 (.089)***	1.83 (.091)***
Treat.		-.01 (.001)***	-.01 (.001)***	-.03 (.004)***	-.04 (.006)***	-.05 (.006)***
F.U.		-.01 (.002)***	-.01 (.002)***	-.01 (.002)**	-.006 (.003)	-.005 (.003)
ADM.			.21 (.100)*	.17 (.097)	.17 (.097)	.047 (.106)
PD.			.41 (.100)***	.42 (.098)***	.38 (.107)***	.40 (.107)***
Treat.*PD.					.007 (.008)	.003 (.007)
F.U.*PD.					.000 (004)	.001 (.004)
Treat.*ADM.						.02 (.007)**
F.U.*ADM.						-.002 (.004)
Random part						
$\sigma_{e}^{2}$	.492 (.022)***	.413 (.018)***	.413 (.018)***	.260 (.013)***	.260 (.013)***	.260 (.013)***
$\sigma_{u0}^{2}$	.364 (.050)**	.384 (.050)***	.324 (.044)***	.348 (.050)***	.349 (.050)***	.343 (.049)***
$\sigma_{u1}^{2}$				.001 (.0003)***	.001 (0003)***	.001 (.000)***
$\sigma_{u2}^{2}$				n.e.	n.e.	n.e.
σ*_u_*_01_				-.007 (.003)**	-.007 (.003)**	-.006 (.003)*
σ*_u_*_02_				n.e.	n.e.	n.e.
–2ll	2796.710	2621.049	2598.202	2367.484	2366.548	2359.320

Outcome is depression scale on SCL-90-R. Est., estimated value; s.e., standard error; ADM., antidepressant medication present or not present during treatment; PD., comorbid personality disorder present or not present; Int., intercept; Treat., estimated change in outcome per month in treatment. F.U., estimated change in outcome per month in follow-up; Treat*ADM., interaction between change in outcome during treatment and use of ADM; F.U.*ADM., interaction between change in outcome during follow-up and use of ADM; Treat*PD., interaction between change in outcome during treatment and comorbid PD; F.U.*PD., interaction between change in outcome during follow-up and comorbid PD; n.e., not estimated. $\sigma_{e}^{2}$ = repeated measures variance. $\sigma_{u0}^{2}$ = intercept variance (beteween subjects). $\sigma_{u1}^{2}$ = treatment slope variance (between subjects). $\sigma_{u2}^{2}$ = follow-up slope variance (between subjects). σ*_u_*_01_ = intercept–treatment slope covariance. σ*_u_*_02_ = intercept–follow-up slope covariance * = significant at.05. ** = significant at.01. *** = significant at.001. –2ll = –2 log likelihood deviance test. † = model was specified with fixed and random effects for treatment slope (i.e., between subjects variation was estimated for this time phases), but only fixed effect (i.e., means) for follow-up due to lack of significant between-person variance in this slope.

**Table 2:** Results from multilevel analysis of development of interpersonal problems (IIP-64) during treatment and follow-up.

Model	Model 0: Intercept only	Model 1: + time	Model 2: + ADM, PD	Model 3†: + time random	Model 4†: + interaction PD	Model 5†: + interaction ADM
Fixed part	Est. (s.e.)	Est. (s.e.)	Est. (s.e.)	Est. (s.e.)	Est. (s.e.)	Est. (s.e.)
Int.	1.392 (.036)***	1.484 (.038)***	1.227 (.058)***	1.291 (.058)***	1.286 (.060)***	1.320 (.060)***
Treat.		-.004 (.0007)***	-.005 (.0007)***	-.012 (.002)***	-.011 (.003)***	-.015 (.003)***
F.U.		-.006 (.001)***	-.006 (.001)***	-.003 (.001)**	-.005 (.002)***	-.006 (.002)***
ADM.			.088 (.068)	.067 (.067)	.066 (.067)	-.008 (.071)
PD.			.391 (.068)***	.408 (.068)***	.417 (.072)***	.432 (.071)***
Treat.*PD.					-.002 (.004)	-.004 (.004)
F.U.*PD.					.003 (.002)	.003 (.002)
Treat.*ADM.						.009 (.004)**
F.U.*ADM.						.002 (.002)
Random part						
$\sigma_{e}^{2}$	.132 (.006)***	.116 (.005)***	.116 (.005)***	.076 (.004)***	.075 (.004)***	.075 (.004)***
$\sigma_{u0}^{2}$	.192 (.024)***	.201 (.025)***	.160 (.020)***	.173 (.023)***	.173 (.023)***	.170 (.022)***
$\sigma_{u1}^{2}$				.0003 (.000)***	.0003 (.000)***	.0002 (.0001)***
$\sigma_{u2}^{2}$				n.e.	n.e.	n.e.
σ*_u_*_01_				-.002 (.001)*	-.002 (.001)*	-.001 (.001)
σ*_u_*_02_				n.e.	n.e.	n.e.
–2ll	1342.405	1216.036	1181.819	959.607	956.998	945.484

Outcome is inventory of interpersonal problems 64 (IIP-64). Est., estimated value; s.e., standard error; ADM., antidepressant medication present or not present during treatment; PD., comorbid personality disorder present or not present; Int., intercept; Treat., estimated change in outcome per month in treatment; F.U., estimated change in outcome per month in follow-up; Treat*ADM., interaction between change in outcome during treatment and use of ADM; F.U.*ADM., interaction between change in outcome during follow-up and use of medication; Treat*PD., interaction between change in outcome during treatment and comorbid PD; F.U.*PD., interaction between change in outcome during follow-up and comorbid PD; n.e., not estimated. $\sigma_{e}^{2}$ = repeated measures variance. $\sigma_{u0}^{2}$ = intercept variance (beteween subjects). $\sigma_{u1}^{2}$ = treatment slope variance (between subjects). $\sigma_{u2}^{2}$ = follow-up slope variance (between subjects). σ*_u_*_01_ = intercept–treatment slope covariance. σ*_u_*_02_ = intercept–follow-up slope covariance. * = significant at.05. ** = significant at.01. *** = significant at.001. –2ll = –2 log likelihood deviance test. † = model was specified with fixed and random effects for treatment slope (i.e., between subjects variation was estimated for this time phases), but only fixed effect (i.e., means) for follow-up due to lack of significant between-person variance in this slope.

First, an intercept-only model (Model 0) was fitted. This model was without explanatory variables and contained a fixed and random intercept, estimating the mean of the outcome across timepoints and residuals at the higher (i.e., patient) and lower (i.e., each timepoint within individual) levels. The model was used to calculate intra-class correlations (ICC; i.e., variance between individuals vs. variance within individuals across time), and serve as a benchmark for subsequent model fit evaluation. In the second step we added lower-level explanatory variables (i.e., time slopes) fixed (Model 1). Variance in the slopes was fixed to zero, so the model only estimated means of the symptom change for each slope. Thus, ‘treatment’ and ‘follow-up’ were added as variables without random effects. The LRT test showed a significant improvement in model fit for both Dep-SCL-90-R (Δχ^2^ (2) = 175.661, *p <*.001) and IIP-64 (Δχ^2^ (2) = 126.369, *p <*.001).

According to Hox et al. (41) it is recommended to add fixed regression coefficients before variance components in the model building process. Thus, in the third step we added higher-level explanatory variables (i.e., between-person variables), fixed (Model 2). ADM (‘not present’ coded as 0, ‘present’ coded as 1) and comorbid personality disorder (PD; ‘not present’ coded as 0, ‘present’ coded as 1) were added as explanatory variables for overall symptom level. The LRT test showed a significant improvement in model fit for both Dep-SCL-90-R (Δχ^2^ (2) = 22.847, *p <*.001) and IIP-64 (Δχ^2^ (2) = 34.217, *p <*.001).

In the fourth step we added random effects to the time-slopes (Model 3), allowing each individual to have varying rates of change. When running this model, the results revealed that there was no significant between-person variance for the follow-up slope for the Dep-SCL-90-R and IIP-64. The model was thus respecified without random slope for follow up. Thus, the final model was specified with fixed and random effects for ‘treatment’, but only fixed effects for ‘follow-up’ (see Model 3 in **Table 1** for Dep-SCL-90-R, and **Table 2** for IIP-64). The LRT test showed a significant improvement in model fit for both Dep-SCL-90-R (Δχ^2^ (2) = 230.718, *p <*.001) and IIP-64 (Δχ^2^ (2) = 222.212, *p <*.001).

In the fifth step we added two-level interactions between time-slopes and PD (see Model 4 in **Table 1** for Dep-SCL-90-R, and **Table 2** for IIP-64). The LRT test showed that there was no significant improvement in model fit when adding this parameter for Dep-SCL-90-R (Δχ^2^ (2) = .936, *p = .*626) or IIP-64 (Δχ^2^ (2) = 2.609, *p <*.271). However as our research question was about assessing differences between ADM users and nonmedicated patients controlling for PD, we retained the parameter in the model as a control variable before adding ADM interactions to the model.

In the sixth step we added two-level interactions between time-slopes and ADM (see Model 5 in **Table 1** for Dep-SCL-90-R, and **Table 2** for IIP-64). The LRT test showed a significant improvement in model fit for both Dep-SCL-90-R (Δχ^2^ (2) = 7.228, *p = .*027) and IIP-64 (Δχ^2^ (2) = 11.514, *p = .*003). We accordingly retained the model for both outcomes.

As there were no significant interactions between PD and time-slopes for any of the outcomes (see results), we did not add three way interactions between time-slopes, ADM and PD. We also estimated effect sizes for symptom reduction from start to end of treatment, end of treatment to follow-up, and start to follow-up for medicated and nonmedicated patients. According to Cohen (43), we used the standard deviations for the Norwegian reference samples of.52 for Dep-SCL-90-R (35), and.59 for IIP-64 (39). Analyses were conducted using SPSS v.29.

## 3 Results

### 3.1 Descriptives

The patients had a mean depression level of 2.17 (*SD* = .74) on Dep-SCL-90-R at start of treatment. A t-test revealed there was no significant difference (*t* (165) = -1.094, *p* = .276) between mean (M) baseline depression levels of ADM users (*M* = 2.22, *SD* = .75) and nonmedicated patients (*M* = 2.09, *SD* = .71). Of the 166 patients, 95.1% had a depression score above the.95 cut-off for clinical severity on the SCL-90-R depressive scale (44). Of the total sample of 166 patients, 59% (n=98) did no longer have a depression diagnosis at the end of treatment. The patients had a mean level of interpersonal problems of 1.6 (*SD* = .51) at start of treatment. A t-test revealed there was no significant difference (*t* (158) = -1.478, *p* = .141) between mean levels of interpersonal problems for ADM-users (*M* = 1.66, *SD* = .46) and non-medicated patients (*M* = 1.54, *SD* = .55) at baseline.

### 3.2 Changes in depressive symptoms

**Figure 2:**
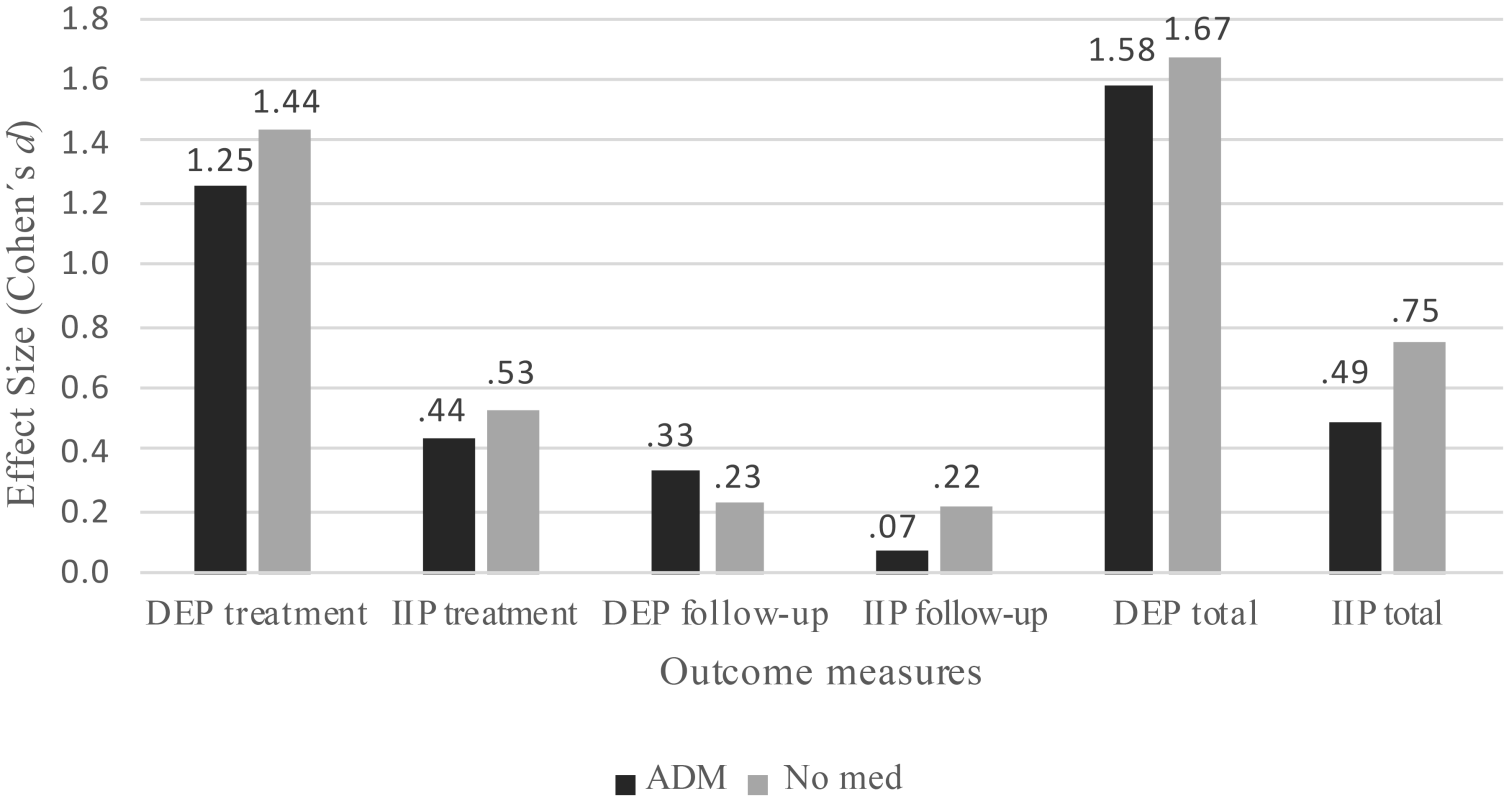
Cohens’ d effect sizes for nonmedicated and medicated patients. DEP, Depression scale of Symptom Check List-90-Revised; IIP, Inventory of Interpersonal Problems-64; ADM, Patients using antidepressant medication; No med, nonmedicated patients.

**Table 1** shows the results of the MLM for depressive symptoms. The intercept-only model (Model 0) showed the ICC was.425, indicating that 42.5% of the total variance of depression symptoms was variance at level 2 (i.e, between individuals) while 57.5% was variance at level 1 (within individuals across time). The final model (Model 5) predicted an initial level of depressive symptoms of 1.8 ( $\hat{y}$ = 1.83, *p* <.001) on the dep-SCL-90-R, with a significant symptom reduction per month during treatment of.05 ( $\hat{y}$ = -.05, *p* <.001). This corresponded to an effect size of 1.35 (Cohens *d*) during treatment. There was no significant change during follow-up ( $\hat{y}$ = -.005, *p* =.172), indicating stability of treatment gains 2.5 years after treatment ended. The MLM model further corroborated the findings of the t-test indicating no significant difference in depressive symptoms between nonmedicated patients and ADM users at intercept ( $\hat{y}$ = .047, *p* = .658). Patients with comorbid PD had significantly higher symptom levels at intercept ( $\hat{y}$ = .40, *p* <.001). Patients using ADM had lower rate of symptom reduction per month during treatment than nonmedicated patients, as indicated by the significant interaction effect between ADM and treatment ( $\hat{y}$ = .02, *p* = .008). The effect size of symptom reduction during treatment was 1.44 for nonmedicated patients, and 1.25 for patients using ADM (**Figure 2** presents effect sizes for ADM users and nonmedicated patients during treatment and follow-up). There was no interaction effect between follow-up and ADM ( $\hat{y}$ = -.002, *p* = .582).

Patients with comorbid PD did not have different rates of change than patients without PD, as indicated by the non-significant interaction during treatment ( $\hat{y}$ = .003, *p* = .693) and follow-up ( $\hat{y}$ = .001, *p* = .798). There was also a significant covariance between the initial symptom level and symptom change during treatment ( $\hat{y}$ = -.006, *p* = .013), indicating patients who started out with higher initial symptom levels had steeper symptom decrease than patients with lower symptom levels.

A supplemental t-test revealed there was no significant difference between ADM users (*M* = .76, *SD* = 1.10) and nonmedicated patients (*M =* 1.03, *SD* = 1.07) on total symptom change during treatment (*t* (125) = 1.40, *p* = .163).

### 3.3 Changes in interpersonal problems

**Table 2** shows the results of the MLM for interpersonal problems. The intercept-only model (Model 0) showed an ICC of.593, indicating 59.3% of the total variance of interpersonal problems was variance between individuals, while 40.7% was variance within individuals across time. The final model (Model 5) predicted an initial level of interpersonal problems of 1.3 ( $\hat{y}$ = 1.32, *p* <.001), a significant reduction during treatment of.02 per month ( $\hat{y}$ = -.015, *p* <.001), and a significant reduction of.006 per month during follow-up ( $\hat{y}$ = -.006, *p* <.001). This corresponded to an effect size of.47 during treatment, and.15 for the follow-up period. There was no difference in interpersonal problems between nonmedicated patients and ADM users at baseline ( $\hat{y}$ = -.008, *p* = .911), but patients with comorbid PD had higher levels of interpersonal problems ( $\hat{y}$ = .43, *p* <.001). Patients using ADM had less reduction of interpersonal problems during treatment than nonmedicated patients, as shown in the significant interaction effect between ADM and treatment ( $\hat{y}$ = .01, *p* = .006). The effect size of symptom reduction during treatment was.53 for nonmedicated patients, and.44 for patients using ADM (see **Figure 2**). There was no interaction effect during follow-up for ADM ( $\hat{y}$ = .002, *p* = .261). Patients with comorbid PD did not have different rates of change than patients without PD, as shown in the non-significant interactions during treatment ( $\hat{y}$ = -.004, *p* = .248) and follow-up ( $\hat{y}$ = .003, *p* = .151).

A supplemental t-test revealed there was no significant difference between ADM users (*M* = .32, *SD* = .52) and nonmedicated patients (*M = .*33, *SD* = .68) on total change during treatment (*t* (127) = .12, *p* = .906).

## 4 Discussion

In this study we explored symptom change in open-ended psychotherapy for depressed patients comparing nonmedicated patients and ADM users. The samples’ initial mean on Dep-SCL-90-R of 2.2, and the high percentage (95.1%) who scored over the.95 cut-off score for clinical severity, indicated a higher degree of depression compared to other mental health outpatient samples in Norway reporting a mean depression score of 1.83 (44). Still, there was a primary diagnostic remission rate of 59% during treatment and an overall effect size of 1.35 for depressive symptoms and.47 for interpersonal problems. For interpersonal problems symptom scores also continued to decrease during follow-up (*d* = .15). Overall, ADM users and nonmedicated patients did not differ in depressive symptoms nor in interpersonal problems at pre-treatment, whereas patients with comorbid PD experienced more distress on both outcome measures. Although the rates of change did not significantly differ between patients with and without PD, it is noteworthy that both groups experienced substantial improvement. This is in line with previous research indicating psychotherapy is a viable alternative for patients with persistent depression both with and without comorbid PD (45).

Nonmedicated patients exhibited greater progress in both depressive symptoms and interpersonal problems compared to those using ADM. This may seem paradoxical given findings indicating combination of psychotherapy and ADM outperforms monotherapy (1–6). One explanation for this could be that ADM had suppressed an initial higher level of depressive symptoms for ADM users resulting in non-significant symptoms differences at start of treatment. However, this does not account for the difference between groups during treatment. On the other hand, our results are in line with research indicating that long term use of ADM and previous treatment with ADM may induce or worsen depressive symptoms (10, 11), which in turn increases the risk of chronicity and vulnerability to depressive disorders (14, 46). This phenomenon, known as iatrogenic comorbidity, may result in a lower level of symptom change when compared to nonmedicated patients. Another consequence of ADM use could be that it makes patients less accessible to the psychotherapeutic interventions, and thus, may hinder patients in fully engaging in the psychotherapeutic processes offered to them (e.g., 16). The fact that 64% of the ADM group had been using ADM during the last two years prior to treatment (compared to 28% in the nonmedicated group) indicates that ADM users had been using medication for a long time. Furthermore, t-tests revealed no significant difference on total change for both depressive symptoms and interpersonal problems, and ADM users were almost twice as long in treatment as nonmedicated patients (see **Figure 1**). The results thus indicated ADM users needed longer time in treatment to achieve similar outcomes as nonmedicated patients for both problem domains. Accordingly, iatrogenic comorbidity may have contributed to less or slower improvement form ADM users compared to nonmedicated patients. An implication of this could be that long term ADM users with depression may need more time in therapy compared to non-medicated patients or that one should consider whether continued use of ADM is productive.

## 5 Limitations

Despite the strengths of this study, such as high ecological validity using a typical outpatient sample with few exclusion criteria and a high level of clinical disturbance, there are potential limitations that should be taken into account. First, we do not have a design that permits us to draw causal conclusion about the effects of medication and/or psychotherapy in this study, and results should thus be interpreted with caution. Given the non-randomized design, it is possible that confounding variables such as differences in symptom severity, treatment expectations, or prior treatment failures could have impacted the results. The fact that these patients receive relatively long treatments as part of the public mental health service in Norway may limit the generalization to other populations but may on the other hand provides with a ground to study real life change in this group of patients that we seem to encounter in every country. Some of the patients on ADM during treatment had not been using it before (n = 30), while most had been on ADM also before treatment (n = 59). Differences in prior use of medication could have influenced results. Also, we did not have information on whether patients on ADM adhered to their treatment regimens, and we did not control the actual serum levels of the ADM, which may further limit our conclusions.

## Data Availability

The data analyzed in this study is subject to the following licenses/restrictions: The data that support the findings of this study are not publicly available due to privacy or ethical restrictions. Pending approval from the treatment facility that all data are made anonymous and in compliance with GDPR and other local regulations, the data may be made available on request from the corresponding author. Requests to access these datasets should be directed to andrhos@uio.no.
