# Supplementary material for: Use of antidepressant medication is associated with slower response to open-ended psychotherapy for depressed patients
Source: Front Psychiatry. 2025 Sep 1;16:1503848. doi: 10.3389/fpsyt.2025.1503848 (PMC12433994; doi:10.3389/fpsyt.2025.1503848)
Supplement: Supplementary file 1 [file Table1.docx]

**Supplemental materials**

**Detailed description of data structure**

Each patient was assessed at 11 occasions (i.e., start of treatment (T1), 3rd, 12th, 20th, 40th, 60th and 80th sessions, as well as end of treatment (T2), six month follow up (T3), one-year follow-up (T4) and two-and-a-half-year follow-up (T5). The dataset (N=166) was organized in long format so that there was a line of data for each patient at each measurement occasion and corresponding time (i.e., number of months) for each occasion. All patients had the same occasions (i.e., T1, 3rd session, 12th session and so on), but the corresponding months that had elapsed at each occasion varied. Thus, each patient had an individual timeline measured in months, were each occasion corresponded to the number of months that had passed since start of treatment. As patients had been in open ended treatment patients could have fewer sessions than measurement occasions (e.g., a patient could end treatment after 40 sessions and thus have missing values on sessions 60 and 80). In these cases, the time score of the last session was imputed for the subsequent sessions, and outcomes were left missing for these occasions. For instance, if a patient ended treatment after the 40th session and 13.8 months had passed at his point, 13.8 was entered for the 60th and 80th occasion (see supplemental table S1).

**Table S1**

*Illustration of data structure for piecewise model with two phases (treatment, and follow-up).*

| Id | Occasion | Treat/FU | Months | Treat | FU | Dep |
| --- | --- | --- | --- | --- | --- | --- |
| 1 | T1 | Treat | 0 | 0 | 0 | 2.08 |
| 1 | 3^rd^ | Treat | 1.5 | 1.5 | 0 | 2.31 |
| 1 | 12^th^ | Treat | 4.1 | 4.1 | 0 | 1.38 |
| 1 | 20^th^ | Treat | 6.4 | 6.4 | 0 | 1.62 |
| 1 | 40^th^ | Treat | 13.8 | 13.8 | 0 | .77 |
| 1 | 60^th^ | Treat | 13.8 | 13.8 | 0 | - |
| 1 | 80^th^ | Treat | 13.8 | 13.8 | 0 | - |
| 1 | T2 | Treat | 14.5 | 14.5 | 0 | .62 |
| 1 | T3 | FU | 20.6 | 14.5 | 6.1 | 1.15 |
| 1 | T4 | FU | 27.4 | 14.5 | 12.9 | .85 |
| 1 | T5 | FU | 42.2 | 14.5 | 27.7 | 1.23 |
| 2 | T1 | Treat | 0 | 0 | 0 | 3.38 |
| 2 | 3^rd^ | Treat | 2.9 | 2.9 | 0 | 2.77 |
| 2 | 12^th^ | Treat | 7.7 | 7.7 | 0 | 1.92 |
| 2 | 20^th^ | Treat | 12.6 | 12.6 | 0 | 2.92 |
| 2 | 40^th^ | Treat | 19.9 | 19.9 | 0 | 1.77 |
| 2 | 60^th^ | Treat | 26.8 | 26.8 | 0 | 2.54 |
| 2 | 80^th^ | Treat | 30.1 | 30.1 | 0 | 1.69 |
| 2 | T2 | Treat | 91.7 | 91.7 | 0 | 1.0 |
| 2 | T3 | FU | 101.1 | 91.7 | 9.4 | 1.15 |
| 2 | T4 | FU | 103.8 | 91.7 | 12.1 | 1.62 |
| 2 | T5 | FU | 118.4 | 91.7 | 26.7 | 2.69 |
| *Note:* Piecewise structure for 2 patients. Patient with id 1 spent a total of 14.5 months in treatment and 42.2 months total. Treatment was ended after 40 sessions. Thus, time score for session 60 and 80 were set to be the same as last session, and outcome for session 60 and 80 were missing. Patent with id 2 spent a total of 91.7 months in treatment and 118.4 months total. The patient kept on having sessions that were not recorded after 80 sessions, thus leaving a considerable timespan between the 80^th^ session and T2. Occasion = occasion for data collection. Treat/FU = indication of whether corresponding occasion and month occurred during treatment or follow-up. Months = total number of months in treatment and follow up. Treat = months in treatment. FU = months in follow-up. Dep = score on depression scale of SCL-90-R. | | | | | | |
